# Epidemiological Characterization of a Directed and Weighted Disease Network Using Data From a Cohort of One Million Patients: Network Analysis

**DOI:** 10.2196/15196

**Published:** 2020-04-09

**Authors:** Kyungmin Ko, Chae Won Lee, Sangmin Nam, Song Vogue Ahn, Jung Ho Bae, Chi Yong Ban, Jongman Yoo, Jungmin Park, Hyun Wook Han

**Affiliations:** 1 Department of Biomedical Informatics CHA University of Medicine Seongnam Republic of Korea; 2 Department of Pathology Medstar Georgetown University Hospital Washington, DC, WA United States; 3 Institute of Basic Medical Sciences School of Medicine CHA University Seongnam Republic of Korea; 4 Department of Ophthalmology CHA Bundang Medical Center Seongnam Republic of Korea; 5 Department of Health Convergence Ewha Womans University Seoul Republic of Korea; 6 Department of Internal Medicine, Healthcare Research Institute Seoul National University Hospital Healthcare System Gangnam Center Seoul Republic of Korea; 7 Department of Microbiology CHA University School of Medicine Seongnam Republic of Korea; 8 Department of Nursing School of Nursing Hanyang University Seoul Republic of Korea

**Keywords:** cohort studies, data science, longitudinal studies, statistical data interpretation, medical informatics

## Abstract

**Background:**

In the past 20 years, various methods have been introduced to construct disease networks. However, established disease networks have not been clinically useful to date because of differences among demographic factors, as well as the temporal order and intensity among disease-disease associations.

**Objective:**

This study sought to investigate the overall patterns of the associations among diseases; network properties, such as clustering, degree, and strength; and the relationship between the structure of disease networks and demographic factors.

**Methods:**

We used National Health Insurance Service-National Sample Cohort (NHIS-NSC) data from the Republic of Korea, which included the time series insurance information of 1 million out of 50 million Korean (approximately 2%) patients obtained between 2002 and 2013. After setting the observation and outcome periods, we selected only 520 common Korean Classification of Disease, sixth revision codes that were the most prevalent diagnoses, making up approximately 80% of the cases, for statistical validity. Using these data, we constructed a directional and weighted temporal network that considered both demographic factors and network properties.

**Results:**

Our disease network contained 294 nodes and 3085 edges, a relative risk value of more than 4, and a false discovery rate-adjusted *P* value of <.001. Interestingly, our network presented four large clusters. Analysis of the network topology revealed a stronger correlation between in-strength and out-strength than between in-degree and out-degree. Further, the mean age of each disease population was related to the position along the regression line of the out/in-strength plot. Conversely, clustering analysis suggested that our network boasted four large clusters with different sex, age, and disease categories.

**Conclusions:**

We constructed a directional and weighted disease network visualizing demographic factors. Our proposed disease network model is expected to be a valuable tool for use by early clinical researchers seeking to explore the relationships among diseases in the future.

## Introduction

Traditionally, clinical researchers have pushed forward to explore a number of risk factors that affect a single disease [1-3], and any diseases previously diagnosed are considered important clinical indicators to predict the disorder under investigation [4,5]. Among various methods for unearthing disease relationships, the concept of network medicine could be better suited to understand health and disease [6-8]. Likewise, a disease network was introduced a decade ago as a useful method to study the complex relationships among diseases [9-17].

Under the assumption that diseases are caused by genetic defects, many disease networks were constructed using genomic data [9,11,14]. For example, Li et al constructed a network to investigate disease relationships according to the genes of their shared pathways [14]. Nonetheless, according to the disease lists of the International Statistical Classification of Diseases, 10th revision (ICD-10), many diseases, such as traumatic bone fracture attributed to a traffic accident, are not related to genetic mutations. As such, genome-based disease networks alone are inevitably limited for accurately representing the complex pathogenesis of the relationships among diseases [18].

Thus, disease networks were later constructed using shared clinical information, such as symptoms and comorbidities [12,17]. Zhou et al generated a symptom-based network of human diseases that was based on the similarity of symptoms [17], whereas Hidalgo et al and Barabási et al constructed a comorbidity network using the Medicare database [7,12]. Because these efforts were focused on demonstrating the relationships among shared diseases or symptoms occurring or present at a single point in time, the networks did not take into account investigations of the temporal order of disease manifestations [19].

Recently, researchers have suggested that disease networks should consider temporal directionality when exploring the connections among diseases [13]. For instance, Jensen et al analyzed temporal disease progression patterns according to disease trajectory using the Danish National Patient Registry. In this study, we constructed a directional and weighted disease network visualizing the effects of demographic factors, such as sex, age, and disease outbreak size, according to the relative risk (RR) among diseases using the National Health Insurance Service-National Sample Cohort (NHIS-NSC) of South Korea, which includes epidemiological time series data of 12 years for approximately 1 million patients.

Finally, we investigated the overall patterns of the associations among diseases; network properties, such as clustering, degree, and strength; and the relationship between the structure of the disease network and demographic factors.

## Methods

### Construction and Visualization of the Disease Network

South Korea is a representative country implementing national health insurance services. The NHIS-NSC contains time insurance information of 1 million out of 50 million Korean (approximately 2%) patients, which was collected between 2002 and 2013. Thus, clinical information can be tracked for 12 years for every patient.

To examine the risk factors for diseases that a patient already had at the beginning of the cohort study, we needed to set an initial period before the main study period to serve as the medical history period. For most chronic diseases, the recommended follow-up interval rarely exceeds 2 years. Therefore, we set the observation period as 2002 through 2003 and the outcome period as 2004 through 2013.

From the sample of 1,016,580 patients who were eligible for National Health Insurance in 2004, we selected 885,125 patients who had at least one record of a medical visit during the aforementioned observation period. We defined this group of patients as the sample cohort. In South Korea, diagnoses are coded in the Korean Classification of Diseases sixth revision (KCD-6), an extension of the ICD-10. The only difference between the KCD-6 and ICD-10 is that the diagnosis codes for Korean medicine are included in the KCD-6 using U20-U99 codes.

To simplify the study, we truncated the KCD-6 codes beyond their third digit, in effect, grouping subcategories of conditions together. In total, the KCD-6, when used between 2002 and 2013, consisted of 2,097 unique diagnoses at the third digit level, and of these, 1,971 diagnoses were included in our data.

Ultimately, we chose only 520 common KCD-6 codes that were the most prevalent diagnoses, covering approximately 80% of the cases for statistical validity.

### Support Offered by the Clinical Evidence From Relationships Among Diseases

All statistical analyses and visualizations were performed using the R package “igraph” (version 3.4.4) and Cytoscape. For calculation of the RR, we sought to obtain *P* values against the null hypothesis, which states that any two diseases present occur independently of one another in the sample cohort. False-discovery rate (FDR) corrections were performed using the Bonferroni method.

Clusters of associated diseases were identified using the random walktrap community detection algorithm [20,21]. This method detects clusters purely according to connectivity (unless specified to use weights) using random walks along edges. The demographic profiling of disease clusters was carried out by pooling the patients identified with at least one of the diagnoses in the cluster.

As a result, patient pools for each cluster are not exclusive but instead overlap somewhat with other clusters. The age distribution of the patient pools was calculated at the beginning of the observation period. An enrichment analysis of the clusters for the KCD categories was performed using the Fisher exact test for adjusted *P* values <.05.

### Topological Characteristics of the Disease Network

In graph theory, the degree of a node is the total number of connections with other nodes. In a directed network, the out-degree of a node is the number of connections with that node as the source, whereas the in-degree of a node is the number of connections with that node as the target. Hence, the degree can be thought of as a measure of the level of disease risk in our network.

In contrast, the strength of a node is the sum of the RRs to achieve connections with other nodes. For example, the out-strength and in-strength of node *i* are defined, respectively, as follows:

s*_out_* (*i*)=∑*_j_* RR*_ij_* (**1**)

s*_in_* (*i*)=∑*_j_* RR*_ji_* (**2**)

where *RR_ij_* is the weight of the edge from node *i* to node *j*, and *RR_ji_* is the weight of the edge from node *j* to node *i*. The out-strength is a measure of the magnitude of disease morbidity, whereas the in-strength is a measure of the magnitude of a disease’s tendency to follow from other diseases.

### Characterization of Large Clusters Throughout Computational Clustering

To calculate the risk ratio from a risk disease D_1_ to an outcome disease D_2_ (D_1_→D_2_), we need to first identify the group of patients at risk of acquiring D_2_. We regarded a patient as being at risk of disease D_2_ if that patient had no record of being diagnosed with D_2_ during the observation period. Patients were considered to be exposed if they had been diagnosed at least once with disease D_1_ during the observation period. The RR of D_1_→ D_2_ was defined using the following formula:

RR=(a / [a + b]) / (c / [c + d]) (**3**)

where a is the number of patients exposed to D_1_ in the initial period and D_2_ in the outcome period; b is the number of patients exposed to D_1_ in the initial period but not exposed to D_2_ in the outcome period; c is the number of patients not exposed to D_1_ in the initial period but exposed to D_2_ in the outcome period; and d is the number of patients not exposed to either D_1_ in the initial period or D_2_ in the outcome period (Table 1).

Since a single misdiagnosis can cause a very large error in the RR value if the numbers in the contingency table are small, we established a minimum size of 947 patients for each group. For example, the diagnosis with the highest prevalence in the initial period was “J20: acute bronchitis,” with 355,045 patients diagnosed at least once in the observation period.

The lowest diagnosis was “R80: isolated proteinuria,” with 947 patients diagnosed during the observation period. Consequently, the at-risk group sizes ranged from 530,080 (885,125 − 355,045) for acute bronchitis to 884,178 (885,125 − 947) for isolated proteinuria.

To select the cutoﬀ value for the RR, we chose the closest integer to the top percentile (ie, the closest integer to x where *P* [RR > x] = .01], which was 4. Therefore, we selected disease relationships with an RR of more than 4 and an FDR-corrected *P* value of <.001 to construct our ﬁnal network.

Accordingly, the prevalence and at-risk group sizes were large enough to accurately determine the RR. Since the self-interaction in this study was not the subject, the total number of theoretical interactions of a total of 520 nodes was found to be 269,880.

**Table 1:** Contingency table for disease-disease risk ratio calculation.

Risk disease in 2002-2003	Outcome disease in 2004-2013	
Exposed	Not exposed	
Exposed	Value^a^	Value^b^
Not exposed	Value^c^	Value^d^

^a^Number of patients exposed to the risk disease (D_1_) and outcome disease (D_2_).

^b^Number of patients exposed to D_1_ but not exposed to D_2_.

^c^Number of patients not exposed to D_1_ but exposed to D_2_.

^d^Number of patients not exposed to either D_1_ or D_2_.

## Results

### Construction and Visualization of the Disease Network

**Figure 1:**
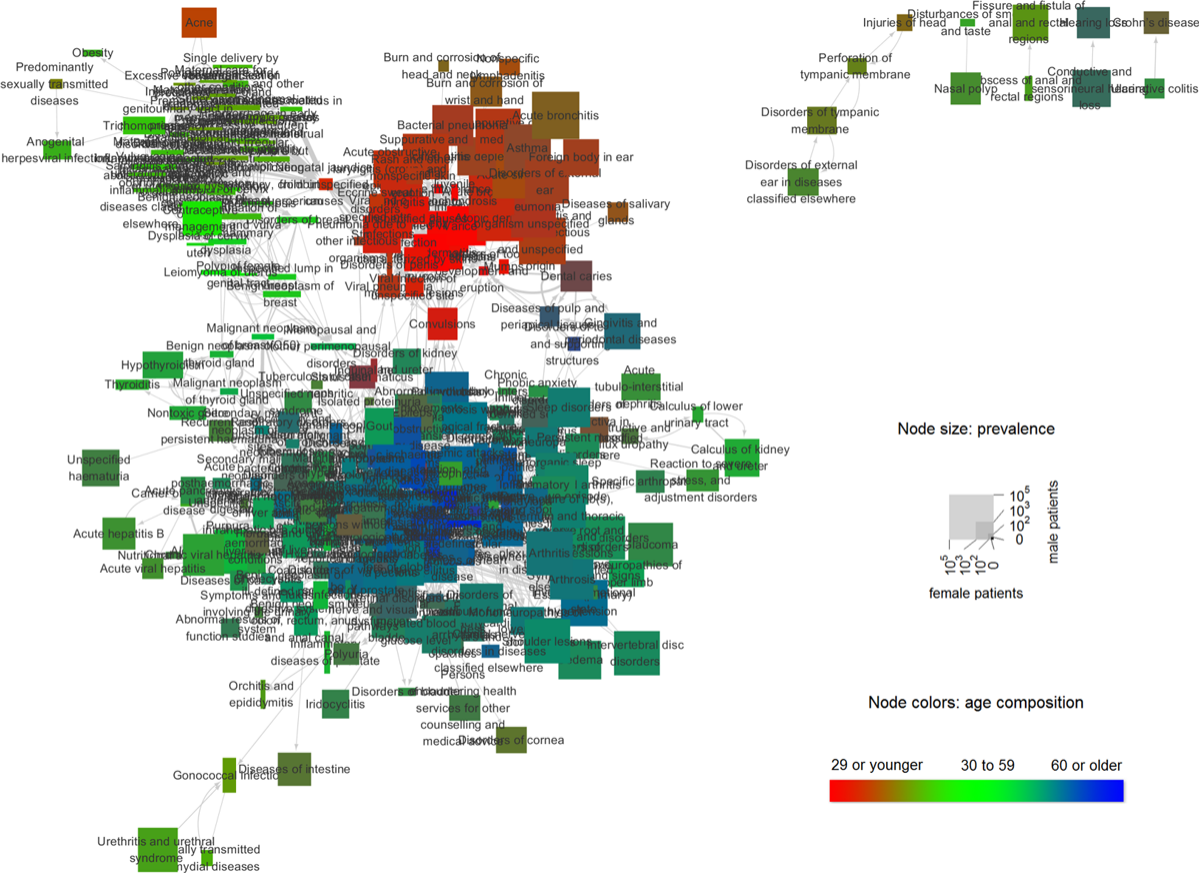
Visualization of the disease network. The network is constructed using the prefuse force-directed layout.

Initially, for the construction and visualization of our final disease network, we selected an RR of more than 4 and an FDR-adjusted *P* value of <.001. As a result, we were able to obtain a disease network with four clusters, 294 nodes, and 3085 edges (Figure 1).

For better clinically intuitive visualization, we designed a visualization scheme such that the color of the disease node would reflect the age of the patient affected with the disease and that the outbreak size would reflect the relative number of patients. The shape of the node was indicated by a rectangle. Node widths represented the number of female patients, whereas the heights represented the number of male patients. For node colors, the intensity of the red channel was proportional to the ratio of patients younger than 30 years, the intensity of the green channel was proportional to the ratio of patients aged between 30 and 59 years, and the intensity of the blue channel was proportional to the ratio of patients aged 60 years or older.

Meanwhile, to indicate the directionality and weight of the node, the edges were represented by arrows and the relative thickness was represented in gray. The number of patients in each sex and age group for each disease was calculated at the beginning of the outcome period based on the included patients’ histories during the observation period.

### Support Offered by the Clinical Evidence From Relationships Among Diseases

To determine whether the correlations among inferred diseases in our disease network model were clinically meaningful, we investigated the available literature concerning the top six disease-disease associations with the highest RR values from the established disease network.

Most results appeared in agreement with previously known associations among diseases (Table 2). A substantial nosologic and biologic overlap exists between bipolar disorder and schizophrenia [22,23]. Further, long-standing hypertension is known to be an important cause and consequence of chronic kidney disease [24]. It is also well-known that anemia develops into chronic kidney disease and portends an unfavorable prognosis [25].

Interestingly, the association between diabetes mellitus in pregnancy and neonatal jaundice was also very high, despite the fact that neonates are never pregnant. This outcome is possible because diagnoses for infants who are not yet in the national registry are filled out under the mother’s account for insurance [26-28]. Another interesting aspect of our results was the fact that neonatal jaundice and diaper dermatitis were strongly associated with one another, which was not observed in previous epidemiological studies.

**Table 2:** Top relative risk values.

Risk factor disease	Outcome disease	RR^a^	References
Bipolar affective disorder	Schizophrenia	34.4	[22,23]
Chronic kidney disease	Hypertensive renal disease	31.9	[24]
Diabetes mellitus in pregnancy	Neonatal jaundice	29.1	[26-28]
Neonatal jaundice	Diaper dermatitis	28.1	N/A^b^
Chronic kidney disease	Anemia in chronic disease	27.4	[25]
Hemorrhage in early pregnancy	Neonatal jaundice	26.1	[27]

^a^RR: relative risk.

^b^N/A: not applicable.

### Topological Characteristics of the Disease Network

**Figure 2:**
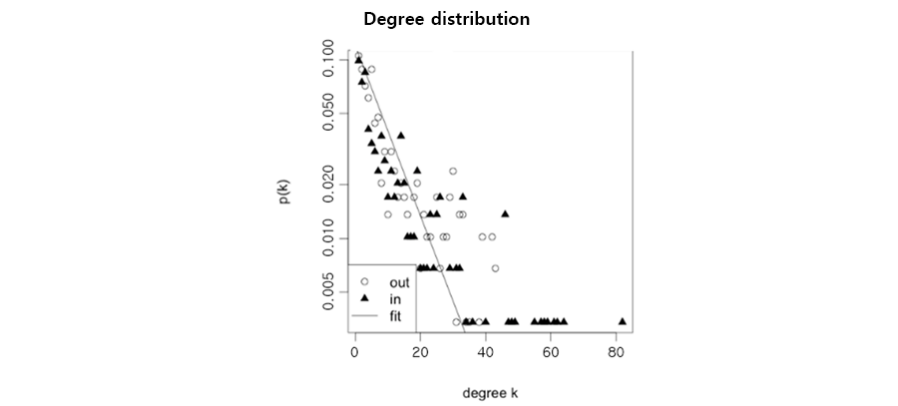
Distribution of the network for in- and out-degrees.

**Figure 3:**
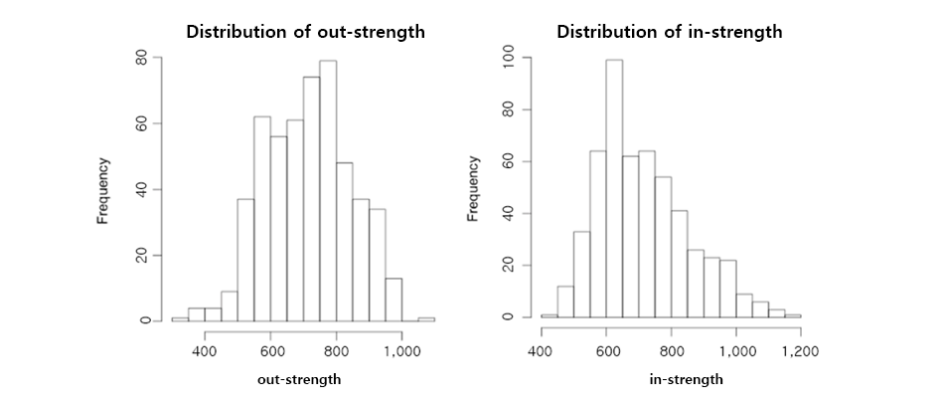
Distribution of the network for in- and out-strength.

We investigated the in- and out-degree distributions of our constructed network. Like many other networks, the in- and out-degrees of our network followed a power-law distribution with a long tail [29] (Figure 2). However, in contrast with the degrees, neither in- nor out-strength followed the power-law distribution (Figure 3).

Table 3 shows the top six diseases with the highest out-degree, in-degree, out-strength, and in-strength results. The top out-degree diseases included diseases that are known to affect many other conditions such as chronic kidney disease and essential hypertension.

The top in-degree diseases are known to be associated with long-term hospitalization or immunocompromise, which are statuses that can arise from various diseases. The top out-degree and top out-strength diseases had considerable overlap, with polyneuropathy, senile cataract, and retinal disorders all being both high out-degree and high out-strength diseases. Patients with these diseases may be at greater risk for developing multiple comorbidities.

The top in-degree and top in-strength diseases included Parkinson disease, chronic kidney disease, anemia in chronic disease, and osteoporosis with pathological fracture. This suggests that many different diseases can have a strong tendency for coverage onto these diseases. Subsequently, we explored the relationships between out-degree and in-degree and between out-strength and in-strength results.

**Figure 4:**
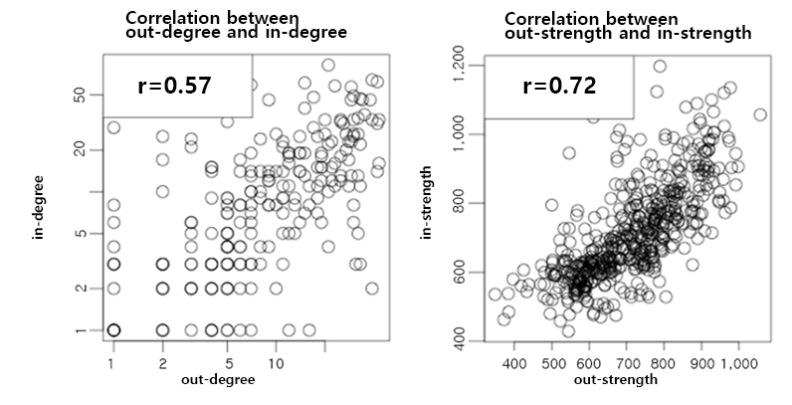
Correlations between in- and out-degrees and in- and out-strengths.

**Figure 5:**
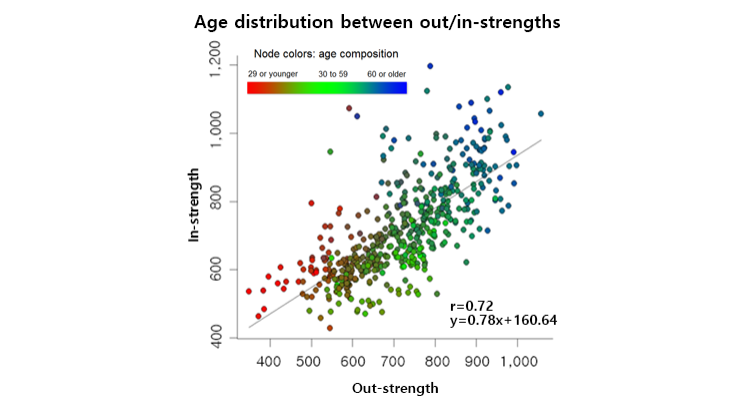
Out- and in-strengths plotted according to the age composition of the patients.

The correlation between the out-strength and in-strength findings (Pearson correlation coefficient: 0.72) was stronger than that between the out-degree and in-degree findings (Pearson correlation coefficient: 0.57) (Figure 4). This means that diseases show strong tendencies to develop from other diseases. For better characterization, we color-coded the diseases in the out-/in-strength plot according to the age composition of the patients (Figure 5). This revealed that mean age was related with positioning along the regression line of the out-/in-strength plot.

**Table 3:** Top out-/in-degree diseases and top out-/in-strength diagnoses.

KCD^a^ code and disease		Degree
**Top out-degree diseases**		
	G63: polyneuropathy	43
	C61: malignant neoplasm of the prostate	43
	H25: senile cataract	43
	H36: retinal disorders	42
	N18: chronic kidney disease	42
	I10: essential hypertension	39
**Top in-degree diseases**		
	G20: Parkinson disease	82
	M80: osteoporosis with pathological fracture	64
	N18: chronic kidney disease	62
	D63: anemia in chronic diseases	61
	A41: sepsis	59
**Top out-strength diseases**		
	G63: polyneuropathy	1057
	H36: retinal disorders	998
	M48: spondylopathies	992
	H25: senile cataract	992
	M81: osteoporosis without pathological fracture	981
	M17: arthrosis of the knee	979
**Top in-strength diseases**		
	G20: Parkinson disease	1197
	N18: chronic kidney disease	1135
	D63: anemia in chronic diseases	1123
	M80: osteoporosis with pathological fracture	1120
	I12: hypertensive renal disease	1100
	H27: disorder of the lens	1089

^a^KCD: Korean Classification of Diseases.

### Characterization of Large Clusters Throughout Computational Clustering

To confirm whether the visually observed clustering in Figure 1 was an artifact of the layout algorithm, we employed a random walktrap algorithm for network clustering [20,21].

**Figure 6:**
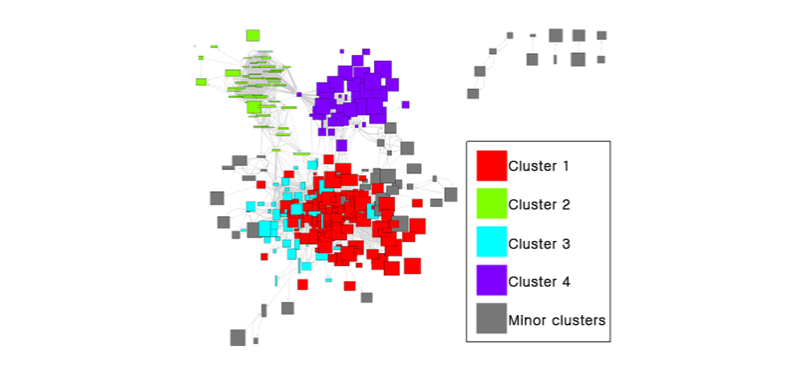
Four major clusters of the network.

A total of 19 clusters were detected, including four large clusters of a size greater than 38 and 15 small clusters of a size less than 13. When we color-coded the network using these four major clusters, we could see that the top right and top left clusters were almost exactly as visualized, but the largest cluster was detected as two large subclusters (Figure 6).

**Figure 7:**
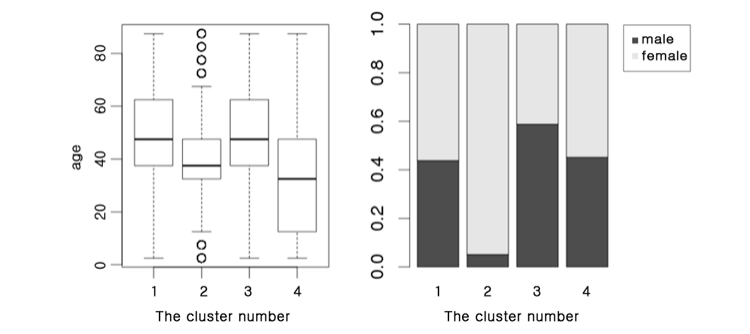
Age distribution and sex ratio for the four major clusters.

This confirmed that disease associations grouped diseases into a few distinct clusters and that this occurred independently of the prefuse force-directed layout. Interestingly, the modularity score for the random walktrap algorithm (0.53) was more than twice the score for the KCD categories (0.24). To see whether the four major clusters actually had the characteristics that we noticed in the visualization, we profiled the clusters with respect to the age distribution and sex ratio of the affected patients (Figure 7).

Patients diagnosed with diseases in clusters 1 and 3 were relatively older (mean age of 47.4 [SD 18.22] years and 48.19 [SD 18.66] years, respectively). The diseases in cluster 2 were dominated by women of reproductive age (the ratio of males to females was 1:18.67; mean age: 39.38 [SD 13.08] years). Cluster 4 included patients who were relatively young, with slightly more females (the ratio of males to females was 1:1.22; mean age: 31.7 [SD 21.56] years).

We profiled the KCD classes of each cluster and performed an enrichment analysis to investigate the types of diseases that were enriched in each cluster (*P*=.05). Although every cluster contained its own disease groups (Multimedia Appendix 1), the enrichment analysis revealed that each of the four major clusters was enriched with nonoverlapping sets of KCD categories (Multimedia Appendix 2). Since each cluster had distinct characteristics, we labeled the major clusters from 1 to 4, according to their most prominent features, as “chronic debilitation,” “women’s disease,” “hemato-oncology,” and “infectious disease” clusters, respectively.

## Discussion

### Principal Findings

In this study, we proposed a comprehensive method for modeling a disease network with directionality and weight of edges using medical claims data. We selected only the most common diagnoses to avoid an overestimation of RR among rare diseases. The ϕ correlation is also useful to avoid overestimation of associations among rare diseases [12], but it is clinically less intuitive and unnecessary for the purpose of studying the overall pattern of common disease associations. Epidemiological factors, such as age and sex, are important inducers of disease development [30-33]; they are, in effect, the most critical clinical factors affecting the prevalence and classification of diseases.

Another purpose of this study was to dissolve these various factors in the disease network and to see how various factors affect the structure and dynamics of the disease network. In addition, these factors were reflected in the visualization of the disease network. In our disease network model, we proposed an intuitive visualization method that maximizes clinical usability.

Nodes indicate the patient outbreak size, and at the same time, represent the relative proportion of width (women) and height (men) in a rectangle. In addition, each node is divided into red for young patients, green for middle-aged patients, and blue for old patients. Conversely, the RR and direction among diseases can be intuitively grasped through the arrow and the thickness of the edge.

As a result, our visualization method of a disease network can help to intuitively identify the direction and RR among diseases and can help to effectively understand the age distribution, sex ratio, and disease outbreak size. The directionality of the disease relationship is a consequence of the study design being longitudinal with a chronological order. Strong RR values support disease association in a chronological order, which is a prerequisite for causality among diseases in clinical research [34]. Because of this, our network can be a starting point to investigate causality among diseases. Here, we examined the literature on disease relationships with high RRs.

### Limitations

The NHIS-NSC includes the proportional stratified sampling data of 1,025,340 patients from among 47,851,928 patients. These patients were randomly extracted by age group, sex, eligibility status, and income level using a proportional stratified sampling method [35]. In general, NHIS-NSC data are representative, but some rare diseases may lose their representativeness owing to the difficulty in obtaining statistical significance. Owing to these limitations, this study excluded disease groups with a small sample size, and rare diseases that have not been assessed in this study are expected to be evaluated in future studies.

As a result, we have shown that our network can provide clues to reveal the causal relationships among diseases. In our network, neonatal jaundice and diaper dermatitis presented a statistically meaningful association (RR=28.1, *P*<.001), but we did not find other supporting evidence of such an association during the literature search. Nonetheless, this does not mean that our network had an incorrect result. Rather, it suggests the possibility of associations that researchers have not yet discovered. In a strict sense, it can be difficult to say that this is a causal relationship because directionality can only be thought of as the natural progression of a disease, the outcome of a treatment, or the process of making a diagnosis.

### Conclusions

During our research, we investigated network topologies, such as degree and strength. Both in- and out-degrees followed power-law distribution like other biological networks; however, strength distributions did not. Since the RR values did not indicate causality, we cannot say that a certain disease is the cause of many other diseases by only looking at out-degrees and out-strengths. Despite this, patients with high out-degree and high out-strength diseases (eg, polyneuropathy, retinal disorders, and senile cataract) are worthy of special attention for secondary prevention purposes. Similarly, diseases with high in-degree and high in-strength findings, such as Parkinson disease, osteoporosis with pathological fracture, and chronic kidney disease, can be seen as common comorbidities of many different diseases.

We found stronger correlations between in- and out-strengths than between in- and out-degrees. Moreover, a stronger risk associated with a disease tended to be related to older affected patients. The association between age and strength suggested that the previously discovered correlation between disease connectivity and mortality could be explained by the phenomenon of increased risk strength.

Through clustering of the network, we found four major disease clusters with distinct demographic characteristics. Interestingly, each cluster was exclusively enriched in KCD categories and had a different mean age and sex ratio. The clustering patterns analyzed using our network suggest that KCD categories, age, and sex have strong influences on disease associations and highlight the importance of demographic factors. Since patients with diseases within a cluster tend to acquire other diseases within the same cluster, we may be able to minimize the onset of comorbidities through patient care by configuring specialty clinics to cater to clusters or subclusters of associated diseases, as is the case with obstetrics and gynecology.

In this regard, our proposed disease network model will likely serve as a valuable tool for early clinical researchers seeking to further explore the relationships of diseases in the future.

For future study attempts, we will take into account the dynamicity of network-considered time order and assess the network collapse point that can affect the overall network structure.

## Appendix

Multimedia Appendix 1 Korean Classification of Diseases composition of disease nodes in each cluster. The composition of each disease cluster in terms of disease categories is shown in this bar chart.

Multimedia Appendix 2 Enrichment analyses revealed that each of the four major communities was enriched with nonoverlapping sets of Korean Classification of Diseases categories.

## Abbreviations

FDR: false-discovery rate
ICD-10: International Statistical Classification of Diseases, 10th version
KCD-6: Korean Classification of Diseases, sixth revision
NHIS-NSC: National Health Insurance Service-National Sample Cohort
RR: relative risk
