# Supplementary figures and images for: Epidemiological Characterization of a Directed and Weighted Disease Network Using Data From a Cohort of One Million Patients: Network Analysis
Source: J Med Internet Res. 2020 Apr 9;22(4):e15196. doi: 10.2196/15196 (PMC7180516; doi:10.2196/15196)

**
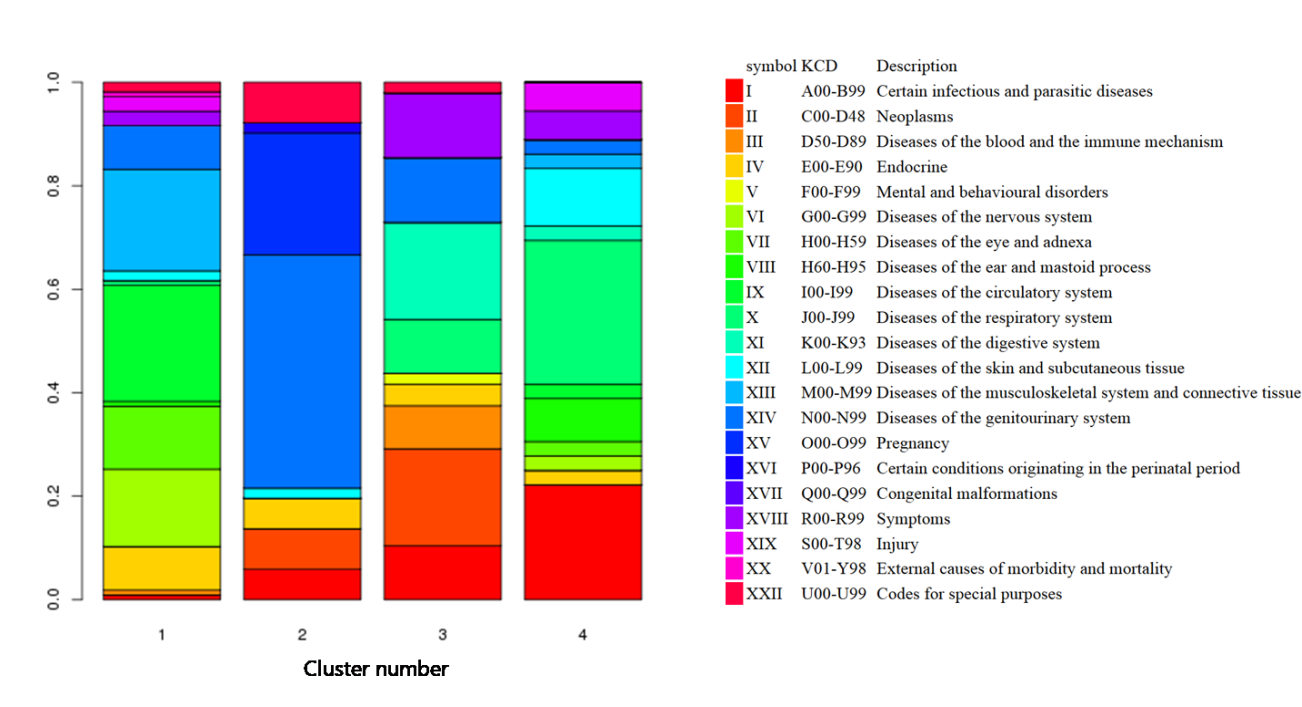
**

Supplement: Multimedia Appendix 1 [file jmir_v22i4e15196_app1.docx]
