# Supplementary material for: Epidemiological Characterization of a Directed and Weighted Disease Network Using Data From a Cohort of One Million Patients: Network Analysis
Source: J Med Internet Res. 2020 Apr 9;22(4):e15196. doi: 10.2196/15196 (PMC7180516; doi:10.2196/15196)
Supplement: Multimedia Appendix 2 [file jmir_v22i4e15196_app2.docx]

| **cluster 1: chronic debilitation cluster** | |
| --- | --- |
| VI | Diseases of the nervous system |
| VII | Diseases of the eye and adnexa |
| IX | Diseases of the circulatory system |
| XIII | Diseases of the musculoskeletal system and connective tissue |
| **cluster 2: women’s disease cluster** | |
| XIV | Diseases of the genitourinary system |
| XV | Pregnancy |
| XXI | tors influencing health status and contact with health services |
| **cluster 3: hemato-oncology cluster** | |
| II | Neoplasms |
| III | Diseases of the blood and the immune mechanism |
| XI | Diseases of the digestive system |
| XVIII | Symptoms |
| **cluster 4: infectious disease cluster** | |
| I | Certain infectious and parasitic diseases |
| X | Diseases of the respiratory system |
| XII | Diseases of the skin and subcutaneous tissue |

**Fisher’s exact test, *adjusted P-value* =.05**
